# Infrared Thermography in Wound Care, Surgery, and Sports Medicine: A Review

**DOI:** 10.3389/fphys.2022.838528

**Published:** 2022-03-03

**Authors:** Jose L. Ramirez-GarciaLuna, Robert Bartlett, Jesus E. Arriaga-Caballero, Robert D. J. Fraser, Gennadi Saiko

**Affiliations:** ^1^Swift Medical Inc., Toronto, ON, Canada; ^2^Division of Experimental Surgery, McGill University, Montreal, QC, Canada; ^3^Centro del Pie del Diabético, San Luis Potosí, Mexico; ^4^Arthur Labatt Family School of Nursing, Western University, London, ON, Canada; ^5^Department of Physics, Ryerson University, Toronto, ON, Canada

**Keywords:** thermography, inflammation, infection, perfusion, surgery

## Abstract

For many years, the role of thermometry was limited to systemic (core body temperature) measurements (e.g., pulmonary catheter) or its approximation using skin/mucosa (e.g., axillary, oral, or rectal) temperature measurements. With recent advances in material science and technology, thermal measurements went beyond core body temperature measurements and found their way in many medical specialties. The article consists of two primary parts. In the first part we overviewed current clinical thermal measurement technologies across two dimensions: (a) direct vs. indirect and (b) single-point vs. multiple-point temperature measurements. In the second part, we focus primarily on clinical applications in wound care, surgery, and sports medicine. The primary focus here is the thermographic imaging modality. However, other thermal modalities are included where relevant for these clinical applications. The literature review identified two primary use scenarios for thermographic imaging: inflammation-based and perfusion-based. These scenarios rely on local (topical) temperature measurements, which are different from systemic (core body temperature) measurements. Quantifying these types of diseases benefits from thermographic imaging of an area in contrast to single-point measurements. The wide adoption of the technology would be accelerated by larger studies supporting the clinical utility of thermography.

## Introduction

The concept of body heat as a marker of disease has a long history in clinical practice. In the time of Hippocrates, fever and chills were known as signs of morbid processes and the hand was used to detect the heat or cold of the human body. Later, with the advent of the Alexandrian School, the clinical exam focus was shifted to pulse measurements. In the Middle Ages, fever again gained prominence as the four humors were assigned the qualities of hot, cold, dry, and moist (Pearce, 2002). Quantitative temperature measurements started at the beginning of the 17th century when several scholars in Britain and Italy independently developed the first thermometers. While it is difficult to establish the priority of the thermometer invention, it is known that Santorio Sanctorius (1561–1636), a physician from Padua, was the first, who developed and applied thermometers to determine patients’ body heat (Santorio et al., 1646). However, only in the late 19th century did the fever thermometer come into general clinical use and found its way into households. It can be attributed primarily to the invention of the mercury thermometer by Aitkin in 1852 and the design of a conveniently portable clinical thermometer by Thomas Clifford Allbutt in 1866 (Pearce, 2003). His 6-inch clinical thermometer measured a temperature in 5 min and replaced a foot-long previously used model, which required 20 min to do so.

For many years, the role of thermometry was limited to systemic (core body temperature) measurements [e.g., pulmonary artery (PA) catheter (Bridges and Thomas, 2009)] or its approximation using skin/mucosa (e.g., axillary, oral, or rectal) temperature measurements. With recent advances in material science and technology, thermal measurements went beyond core body temperature measurements and found their way in many medical specialties. Nowadays, there are multiple types of temperature measurement devices, which are based on electrical (e.g., thermocouples, thermoresistance, thermistors, diodes, or programmable electronic devices), mechanical (e.g., dilation systems, glass thermometers with liquids, bimetallic thermometers), radiometric (e.g., bolometers and devices on quantum principles), and other (color changes, pneumatic, pyrometric, or acoustic/ultrasonic) principles. This article is not intended as a comprehensive review of all these technologies and their clinical applications. Instead, this article focuses primarily on the thermographic imaging modality in wound care, surgery, and sports medicine. However, a brief overview of other thermal modalities is included where relevant for these clinical applications.

## Technology Overview

As mentioned, temperature measurement technologies can be of multiple types. However, they can be split into two major groups: direct and indirect temperature measurements.

### Direct Measurements

Most contact temperature measurement technologies use the direct method of temperature measurement. In a typical scenario, a sensor (which is typically small compared with the body or body part) is placed in contact with the body part and comes in thermal equilibrium with it. Examples of such measurements can be a mercury thermometer or a PA catheter (Bridges and Thomas, 2009).

### Indirect Measurements

Non-contact (remote) temperature measurement techniques do not measure the temperature directly. Instead, they measure energy flow from the object and derive temperature from these measurements (radiometry). Radiometry is based on the fact that all objects with temperatures above 0K emit electromagnetic radiation in a broad range of wavelengths. However, most of the radiation from surrounding objects (e.g., in the 0–100°C temperature range) is coming for wavelengths 3 μm and longer, commonly referred to as thermal infrared, or IR. For example, the peak of radiation from an object at 300K (27°C) is 9 μm. Objects in this temperature range do not emit noticeable radiation in the visible range of the spectrum. Only objects heated 500°C and above emit noticeable radiation in the visible range of the spectrum (i.e., red-hot metal).

An important concept in radiometry is a blackbody, which is an idealized physical body that absorbs all incident electromagnetic radiation, regardless of frequency or angle of incidence. The blackbody also emits 100% of the absorbed energy, thereby having an emissivity of 1.0 or 100%. The emissivity of an object is the ratio of its energy flow to the energy flow of the blackbody at the same temperature. Thus, a 2-D imaging array of thermal IR sensors does not measure temperature directly, but the energy flow, which is emitted by the target. IR sensors typically measure energy flow over a broad range of spectrums. Then, the temperature is derived from these measurements. In particular, the amount of emitted radiation depends upon both the temperature and the emissivity of the material.

For practical purposes, the middle (MWIR, 3–5 μm) and long (LWIR, 8–14 μm) wavelength infrared (IR) spectral ranges are used.

Finally, thermal optical measurements have an established terminology. According to Glückert and Schmidt (2001), while both pyrometry and thermography refer to the remote measurement of temperature based on heat radiation, a pyrometer is a “radiation thermometer,” which provides point like information, while thermography refers to a “temperature picture,” where the information is optically recorded as an entire scene.

## Sensors

The type of sensors involved in temperature measurements depends on the form of temperature measurement (direct or indirect). Below, we will briefly discuss the most common types of sensors for each type of measurement.

### Direct Measurements

Historically, mechanical temperature sensors (e.g., a mercury thermometer) were the only practical way to measure the temperature. Recently, mercury thermometers were mostly removed from clinical practice due to toxicity concerns. Liquid-crystal contact thermography is another type of direct temperature measurement based on mechanical properties (Benbow et al., 1994).

Currently, electrical temperature sensors have mostly replaced mechanical thermometers in clinical use. The two most common types of electrical temperature sensors are resistance temperature detectors and thermocouples.

Both thermocouples and thermoresistive devices are used in clinical applications. For example, Uleberg et al. (2015) used thermocouples and thermistors as an epitympanic sensor in the auditory canal for continuous monitoring of core temperature in severely injured patients. However, historically, thermocouples were mostly used in many applications. Nowadays, thermistors have become the detector of choice for temperatures below 125°C.

### Indirect Measurements

There are two types of sensors used for indirect thermal measurements: non-quantum and quantum (photon) detectors. Non-quantum detectors respond to radiant energy in a way that causes a change of state in the bulk material (i.e., heating the sensing element produces the bolometer effect). They are mostly based on pyroelectric and ferroelectric materials or microbolometer technology. Most IR cameras have a microbolometer-type detector, mainly because of cost considerations. Microbolometers can be created from metal or semiconductor materials. Metal bolometers are produced from thin foils or metal films and usually work without cooling.

Operations of quantum (photon) detectors are based on quantum effects (e.g., excitation of valence electrons to the conduction band), where the state of electrons in a crystal structure changes in reaction to incident thermal photons.

### Form Factors

Existing thermal measurement technologies are analyzed across two interplaying dimensions: contact vs. remote and single or few-point vs. multi-point measurements.

The distinction between few-point vs. multi-point measurements is quite nominal. For clarity, we will consider multi-point measurement devices as those, which have a regular (rectangular) grid of sensors (e.g., mat or IR camera). All other modalities with several sensors (e.g., an insole or sock) will be considered few-point measurements.

#### Contact

As mentioned before, glass thermometers with galinstan and with red or blue alcohol are currently widely used mechanical temperature sensors for axillary temperature measurements in-home and clinical settings. In addition to these traditional contact thermometers, several monitoring contact modalities have been developed including, temperature monitoring wearables and temperature monitoring surfaces (e.g., mats) (Martín-Vaquero et al., 2019).

##### Single or a Few-Point Temperature Monitoring Wearables

Temperature monitoring is a useful tool for many applications ranging from fertility cycle tracking (Zhu et al., 2021) to ulceration prognosis (Houghton et al., 2013). In particular, it could serve as an early warning system in many applications, including the management of open foot ulcers, Charcot foot, and assessment of the risk of re-ulceration. Wearable technologies offer the possibility of continuous temperature monitoring, which may add additional data points and improve compliance. However, data about using wearable technologies for temperature measurements other than wellness applications are scarce. For example, in a recent review of wearable technologies for foot temperature monitoring in diabetic patients (Martín-Vaquero et al., 2019), the authors were able to identify a handful of experimental technologies, which can be used for foot temperature monitoring. The authors hypothesized that the lack of studies may be driven by the fact that the sensor must be very small and comfortable, but still precise enough during several months and after several washes. As an example of such experimental technology, in Hughes-Riley et al. (2017), the authors embedded a thermistor chip into the fibers of a yarn, which can be used to produce a textile or a garment.

The usability study of socks for diabetic foot temperature monitoring was reported in Reyzelman et al. (2018). The bottom of the socks contained six sensors located at the hallux, three metatarsal points, midfoot, and heel. The data was transmitted wirelessly to an app through Bluetooth. Temperature studies conducted on 35 patients with diabetes showed that the sensors used in the socks are reliable and accurate at detecting temperature and the findings matched clinical observations. Patients also reported that the socks were easy to use and comfortable. Users ranked them at a median score of 9 or 10 for comfort and ease of use on a 10-point scale. Finally, the use of insoles to take measures of temperature in diabetic feet to study the etiology of diabetic foot ulceration has also been explored (Torreblanca González et al., 2021). This system has four sensors at four locations in the insole: the hallux, between the first and second metatarsal head, the lateral side of the foot, and the heel. While these wearable technologies seem promissory for ulceration risk reduction, more research is needed before full clinical adoption can be recommended.

##### Multi-Point Temperature Monitoring Surfaces

Tamura et al. (1993) used 16 thermistors PBN-41E to develop a data logger with a memory card to measure temperature while the person is sleeping. One particular form factor suitable for foot temperature monitoring is a smart thermometric foot mat. Frykberg et al. (2017) reported that a smart thermometric foot mat may detect inflammation preceding the development of ulceration at an average of 5 weeks prior to clinical presentation. Because these reports are even scarcer than those of single points, research into this area presents a significant gap in the current literature.

#### Remote (Non-contact)

With recent advances in new materials, pyrometry and thermography have widely been translated into clinical practice:

##### Single-Point Remote Thermometers

Remote thermometers (pyrometers) have been used in medicine for several decades. For, example, tympanic membrane temperature measurements and temporal artery thermometry are considered reliable and accurate measurements that closely resemble the core body temperature (Gasim et al., 2013). In wound care, remote thermometers have been routinely used to document the wound bed and periwound temperatures. The typical form factor here is a small pistol-like handheld device (Sibbald et al., 2021).

##### Multi-Point Thermal Cameras

Thermography has been an experimental modality in medicine for some time. In recent years it has started getting adopted into regular clinical practice. IR camera architecture is very similar to a digital camera. Thermal imaging sensors for biomedical research and clinical applications use low-cost, uncooled focal plane array (FPA) microbolometers operating in the LWIR range. The resolution of thermographic sensors is much lower than normal cameras. Currently, it is in the range of 60 × 80 or 120 × 160 for regular applications and 640 × 480 for higher-end applications.

## Thermographic Physiological Considerations

**FIGURE 1:**
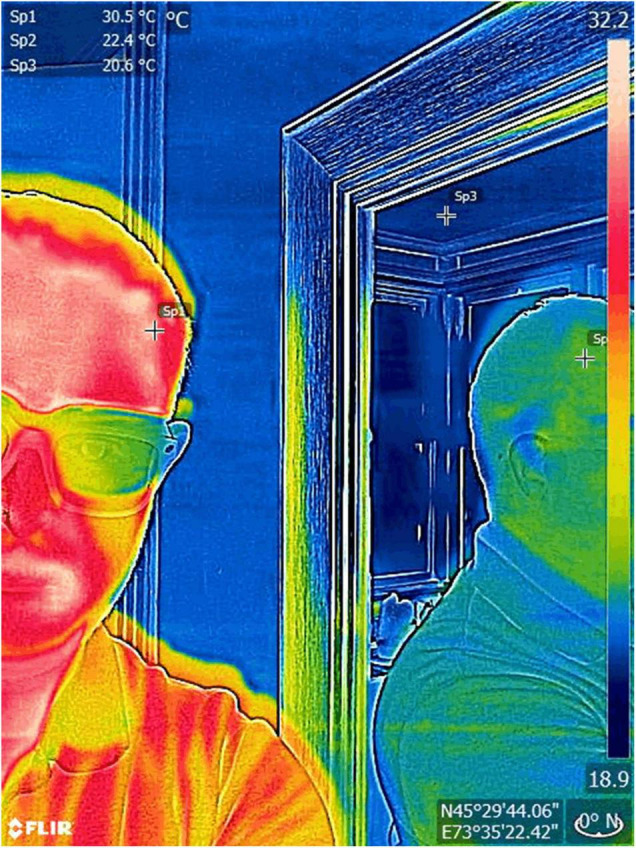
Reflectivity and thermal imaging. The human skin behaves almost like a blackbody with emissivity ranges close to 100%. Thus, this tissue is ideally suited for infrared thermal imaging because the surrounding infrared radiation is absorbed and the output that the camera images and measures correspond almost entirely to the radiation the skin is emitting. In contrast when imaging a reflective surface such as a mirror with an index close to 0%, it is practically impossible to differentiate the radiation emitted by the object and the surrounding radiation scattered by it. In the image above, it can be seen how the skin thermal output (Sp1) can also be seen in a mirror, and how its measurement (Sp2) differs from the output of the surrounding structures (Sp3). Also, note how glasses, despite being transparent for visible light are not under IR. Images, courtesy of JR-G, were acquired using a FLIR One Pro camera.

The skin is the body’s largest organ, covering approximately 2 sq.m. It plays an important role in thermoregulation. Together with adipose tissues, it provides thermal insulation of the body. In addition, it functions as a “heat radiator” system, which is responsible for approximately 90% of body heat loss. The emissivity of human skin is close to the perfect blackbody. It is at least 0.91 in the MWIR range, and even higher (0.97–0.98) in the LWIR range. Because the human body acts almost as a blackbody, it is very well suited for thermographic assessment (Figure 1). In particular, the reflected energy flow, which may impact measurements, is typically quite minimal. However, it is still useful not to perform temperature measurements near hot objects.

### Core Body Temperature

Core body temperature refers to the temperature of the body’s internal organs, such as the heart, liver, brain, and blood. The average normal body temperature is generally accepted as 37°C. However, studies have shown that the “normal” body temperature can have a wide range, from 36.1 to 37.2°C and that it can also be influenced by age, activity, and time of day (Wang et al., 2016).

Core body temperature is tightly regulated by both peripheral and central receptors that are integrated within the hypothalamus (Sessler, 2009). The normal core temperature for an individual can be considered as a “set point” in the thermoregulation system. All the temperature mechanisms continually attempt to bring the body temperature back to this set point. For example, when the body core temperature rises above the critical level of normal body temperature, there is an increase in the rate of heat loss by sweating. Accurate measurements of core body temperature involve invasive medical procedures in hospitals. The PA catheter, which measures blood temperature, is considered the gold standard for measuring core temperatures (Bridges and Thomas, 2009). For non-critical patients, a person’s peripheral body temperature is commonly measured in non-invasive sites such as the mouth, ear, armpit, and rectum because these areas are easily accessible and are believed to provide the best estimation of the core body temperature. However, the accuracy and precision of each of these methods are different. A core temperature measurement can be considered accurate if the mean difference from the PA temperature is ±0.3°C and precise with a standard deviation ranging from 0.3 to 0.5°C (Lawson et al., 2007). A comprehensive summary of accuracy and precision of various non-invasive temperature measurement techniques (the oral, ear-based, temporal artery, the axillary temperature) in intensive care unit (ICU) settings is presented in Bridges and Thomas (2009). The author found that while all methods [with the exception of axillary, which underestimates the PA temperature, were accurate, the precision varied across methods (oral, SD = 0.24–0.6°C; ear-based, SD = 0.4–0.57°C; temporal artery, SD = 0.5–1.1°C; and axillary, SD = 0.16–0.6°C)].

### Skin Temperature

In contrast to the core temperature, the skin temperature falls and rises with the temperature of the surroundings. The skin is an efficient, controlled “heat radiator” system. The flow of blood to the skin is the most effective mechanism for heat transfer from the body core to the environment. The flow of energy to and from the skin determines our sense of hot and cold. For that reason, despite the core body temperature being constant over time, the skin temperature is subjected to changes and is usually lower than the core (Martinez-Jimenez et al., 2021).

Body heat dissipates by radiation, evaporation, convection, and conduction. In normal conditions, radiation is the most significant source accounting for approximately 60% of heat loss (Koop and Tadi, 2021). At rest, evaporation accounts for 22% of heat loss. However, sweating (evaporation) is the primary means of cooling the body during exercise. Different heat loss mechanisms interplay with each other. For example, evaporation and conduction of the air are accelerated by convection. The energy transfer (and skin temperature) is determined also by the temperature and the moisture of the surrounding environment.

The normal skin temperature is about 33°C. However, the skin temperature is not maintained at a constant value across the entire body. Lower temperatures are characteristically observed in proximity to superficial veins, relative to superficial arteries, and over protruding body parts including the toes, fingers, ears, and nose. Similarly, skin surface temperature has been observed to be higher over organs with high metabolic rates rather than those at rest, as well as over muscles rather than tendons or bones (Kanitakis, 2002). The body can vasoconstrict regions of skin (other than chest, neck, and face) exposed to cold temperatures to prevent heat loss. The hand is probably the most active body part in responding to the body’s thermoregulation requirements. In warm conditions, the hand is fully vasodilated, and the fingertips are the warmest areas of the hand. This pattern is reversed when cooling. Vasoconstriction of the hand blood vessels causes the skin temperature of the hand to vary on the order of 8°C. When the hand is cold, it ceases to transfer much body heat to the environment. Because of this, an IR image taken a few minutes after a subject moved from a warm environment (30°C) to a slightly cool (22.6°C) environment, shows that, although the rest of the upper body temperature has not changed much yet, the blood vessels of the hand were already well constricted (Lim, 2020). Likewise, a 3°C difference in finger skin temperature has also been observed in slightly cool environments caused by the difference in muscular exertion between typing vs. holding a computer mouse (Huizenga et al., 2004).

### Absolute vs. Gradient Temperature

Interpreting an absolute skin temperature measurement (especially a single-point measurement) may pose a challenge because of skin thermoregulation. Therefore, a temperature gradient (the difference in temperatures between two points) is a more objective measure, which will depend less on the ambient conditions. Figuratively speaking such an approach is “self-calibrating” where one point (e.g., intact skin) in the same region is selected as a reference. In this case, the positive gradient may be indicative of increased local blood flow (e.g., inflammation), while the negative gradient may be indicative of the reduced blood supply (e.g., obstructed blood flow) (Martínez-Jiménez et al., 2018a; Martinez-Jimenez et al., 2021).

### Wound Bed vs. Peri-Wound

One known problem with thermography is the distortion in the image caused by evaporative water loss in the wound bed. This problem can be solved either by allowing the wound to dry completely (which may delay the timing of the assessment) or by applying a non-permeable covering to the wound bed, which eliminates the problem of evaporation (Cole et al., 1991).

### Viewing Angle

The emissivity and apparent temperature of an object varies with the angle of viewing. Watmough et al. (1970) calculated the emissivity, ε as a function of a refractive index of the material, *n* and viewing angle, φ (see Eq. 1):


(1) $$\in =1-\frac{1}{2}\left\{\frac{\beta -c\,o\,s^{2}\,\varphi }{\beta +c\,o\,s^{2}\,\varphi }\right\}\left[1+{\left[\frac{\beta \,c\,o\,s\,\varphi -s\,i\,n^{2}\,\varphi }{\beta \,c\,o\,s\,\varphi +s\,i\,n^{2}\,\varphi }\right]}^{2}\right]$$

$$\text{Here}\,\beta =\sqrt{n^{2}-s\,i\,n^{2}\,\varphi }.$$

**FIGURE 2:**
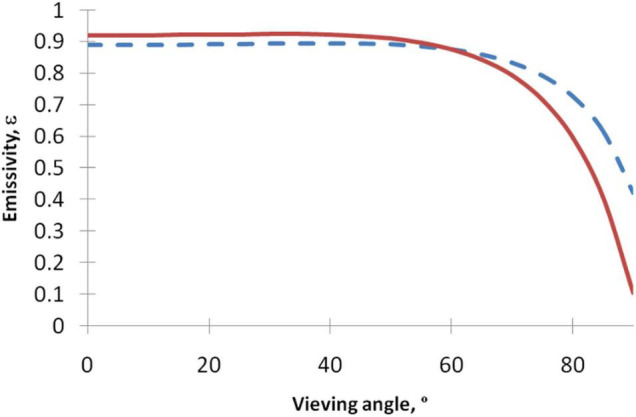
Dependence of the emissivity on the viewing angle for several indexes of refraction. Index of refraction *n* = 1.5 (red solid line), *n* = 2 (dashed blue line). As the angle of viewing increases, the emissivity of the surface is reduced. Thus, for images acquired obliquely, the lower emissivity can lead to a reduction in the apparent temperature of the object in the thermogram.

In Figure 2, we plotted dependence of the emissivity on the viewing angle for several refraction indexes. It can be seen that the emissivity is flat in the range of 0 < φ < 60°. Thus, in practical applications, the angle of viewing with respect to tissue surface should not exceed 60° or ideally 45°, since for larger angles the emissivity of the surface will be significantly reduced. For skin viewed obliquely, the lower emissivity may lead to a reduction in the apparent temperature of more than 4°C, so a significant “hotspot” in this region might be undetected in a thermogram.

### Best Practices

Several practical considerations should be considered for optimal thermal imaging (Moreira et al., 2017). Namely:

• Acclimation – it is recommended to allow some acclimation time before thermal imaging.
• Acute pain – it is not advisable to capture a thermal image immediately following a painful procedure. The associated release of catecholamines with pain is associated with skin vasoconstriction.
• Smokers should be advised not to smoke for an hour in advance of thermal imaging or to use any tobacco or nicotine products to avoid vasoconstriction.
• Energy drinks containing high doses of caffeine and pseudoephedrine that may also cause vasoconstriction.
• Volume depletion – it is not advisable to perform imaging if the patient is volume depleted. A common scenario is a patient with GI upset the day before with associated vomiting and/or diarrhea. If this is the case it is better to allow 24 h for volume repletion.
• Medications – Antihypertensive medications or coffee in moderate doses will not affect thermal images.

## Clinical Utility

Investigation of the utility of thermography in wound care began in the early 1960s when Lawson et al. (2007) used infrared scanning to predict burn depth with an accuracy of 90%, as confirmed by histology. Since then, the utility of thermography has been investigated in many clinical applications. The clinical utility can be split into two primary scenarios:

*Inflammation based (Increased Flow)*

• Infection – powerful predictor of early Surgical Site Infection (SSI).
• Infection – adjunctive predictor of Diabetic Foot Infection (DFI).
• Autoimmune – Charcot Foot (diabetes).
• Autoimmune – Hidradenitis Suppurativa, Lupus Erythematosus, Calciphylaxis, etc.

*Perfusion based (Decreased Flow)*

• Ischemic Diabetic Foot Ulcer (DFU).
• Surgical Free and Rotational Flaps.
• Angioplasty Surveillance.
• Pressure-Induced including Deep Tissue Injury.
• Trauma-Induced.

### Diabetic Foot Ulcers

Diabetic foot ulceration (DFU) is a major complication of diabetes. The annual prevalence of DFU is estimated to be 4–10%, and the lifetime risk of developing these ulcers in people with diabetes is estimated to be anywhere from 15 to 25%. Foot ulceration increases the risk of lower limb amputation, one of the most debilitating complications of diabetes. If untreated, DFU may become infected and require total or partial amputation of the affected limb. More than 50% of non-traumatic lower-limb amputations are attributable to diabetic ulcers (Raghav et al., 2018).

**FIGURE 3:**
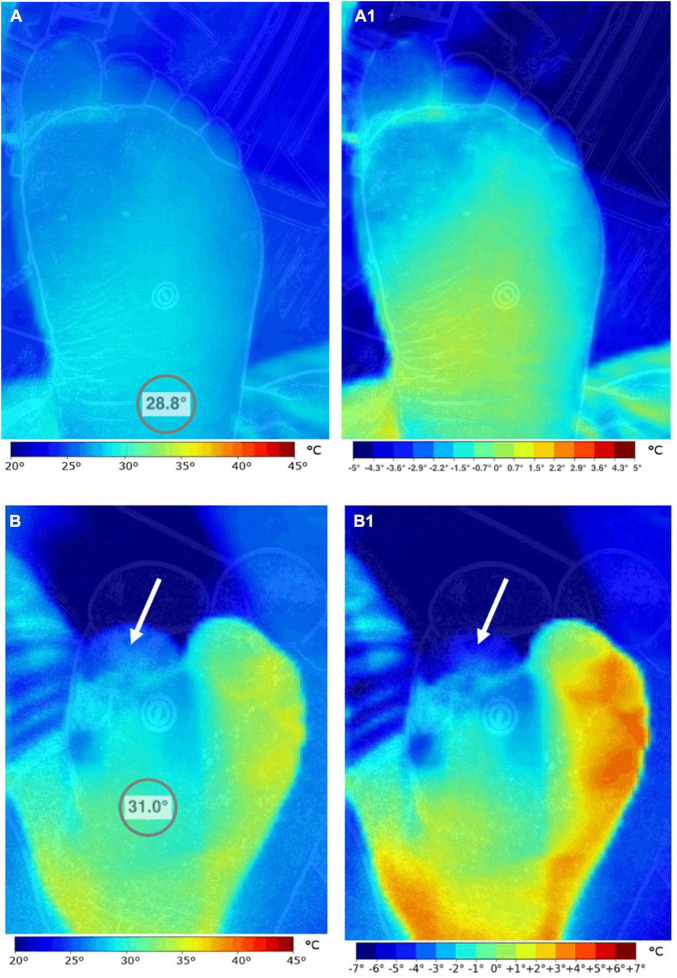
Comparison of the thermographic pattern of two subjects with and without diabetes. The left foot of a healthy subject **(A,A1)** and a patient with diabetes mellitus and vascular complications **(B,B1)** were imaged. Striking differences in the absolute temperature distribution pattern of the foot **(A,B)** can be observed. These differences are magnified when the images are scaled to present temperature gradients **(A1,B1)**. While the healthy control presents a more or less uniform temperature pattern in the hind and mid-foot, the patient with diabetes shows extensive temperature variability that is correlated to microangiopathy and neuropathy. Note that the first toe of the diabetes patient and its medial side show signs of significant vascular compromise (arrows). Images, courtesy of JR-G, were acquired using the Skin and Wound mobile app paired to a Swift Vision camera (Swift Medical Inc., Toronto, ON, Canada).

High throughput screening allows earlier interventions in a patient population that already presents clinically with late-stage complications, significant morbidity, and mortality risk. Thermography has significant potential as an adjuvant technique in diabetic foot assessment. For example, elevated temperature is a reliable marker of inflammation and can thus predict the risk of ulceration, infection, and amputation (Figure 3; Bharara et al., 2012). Similarly, the decreased temperature may be a sign of insufficient blood supply and indicate ischemia (Lin and Saines, 2017).

#### Inflammation Detection

Several approaches based on plantar temperature distributions have been proposed. Benbow et al. (1994) assessed the risk of ulceration and ischemic foot disease based on the mean foot temperature, determined from eight standard sites on the plantar surface. They found that an elevated mean foot temperature was associated with an increased risk for neuropathic foot ulceration. Diabetic patients with normal or low mean foot temperature were at risk for ischemic foot disease.

Another approach is to compare temperature maps of the individual’s contralateral (left foot to their right) sites (an asymmetry analysis). This method has the advantage of being specific to the patient. However, it is dependent on geometrical symmetry between feet (Kaabouch et al., 2010). Preventive care is recommended when a patient is observed with temperature asymmetry exceeding 2.2°C for at least two consecutive days between contralateral sites. Using a remote temperature monitoring mat and the 2.2°C asymmetry threshold, Frykberg et al. (2017) predicted 97% of all non-acute plantar DFU on average 35 days before clinical presentation with a specificity of 43%. Nagase et al. (2011) identified 20 patterns of thermal distribution to aid in diabetic foot assessment and surgical procedures. Bharara et al. (2010) proposed a wound inflammatory index or temperature index for diabetic foot assessment, which is based on the difference in mean foot temperature and the wound bed, the area of the wound bed, and the area of the isotherm (highest or lowest temperature area). Finally, Lavery et al. (2019), in a multicenter study on 129 patients in remission, found that unilateral once-daily foot temperature monitoring can predict 91% of impending non-acute plantar foot ulcers on average 41 days before clinical presentation.

#### Infection Detection

In addition to detecting inflammation, the authors in a case study (Oe et al., 2012) found that thermography may predict osteomyelitis, a severe complication of the diabetic wound before visible signs of infection are shown. Unfortunately, beyond this report, there is a scarcity of evidence to support active detection of infection in DFU through thermography.

#### Blood Perfusion Assessment

**FIGURE 4:**
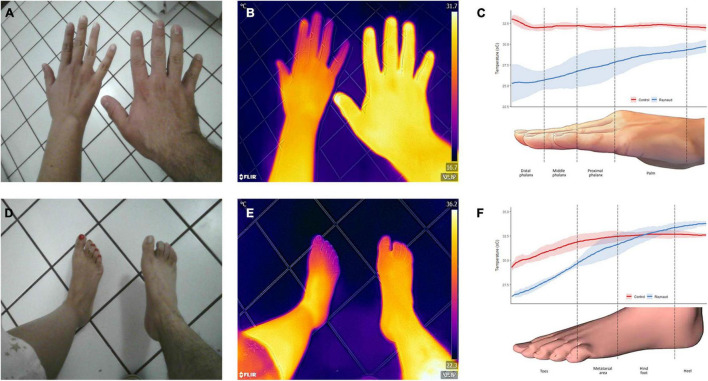
Thermographic assessment of a patient with Raynaud’s phenomenon. Clinical photographs **(A,D)** and infrared thermal images **(B,E)** of the hand and feet of a patient with Raynaud’s phenomenon (left) and a healthy subject (right) were obtained. Color gradients of the heat maps are shown in the far-right side of **(B,E)**. Purple/blue indicates a lower temperature, while yellow/white indicates higher temperatures. Analysis of the temperature distribution across the different structures are presented in **(C,F)**. The patient’s temperature was significantly lower to the control and exhibited a higher degree of variation, as demonstrated by wider confidence intervals. The solid line represents the mean temperature, and the shaded area, its 95% CI. Images, courtesy of JR-G, were acquired using the Skin and Wound mobile app (Swift Medical Inc., Toronto, ON, Canada) paired to a FLIR One Pro camera. Analysis of the images was performed in Swift’s dashboard.

Astasio-Picado et al. (2019) in a study on a group of 277 patients with diabetic pathology found that there were lower temperatures under the 1st metatarsal head, the 5th metatarsal head, the heel, and the pulp of the big toe of both left and right feet of patients in the neuropathy, vasculopathy, and neurovasculopathy groups, relative to the group with neither pathology. In Seifalian et al. (1994), the authors compared Laser Doppler Imaging vs. Laser Doppler Flowmetry vs. Thermographic Imaging for blood perfusion assessment of the skin. They found *r* = 0.577 (*p* < 0.01, *n* = 38) correlation between the thermographic imager and laser Doppler imager in seven normal volunteers. They also found *r* = 0.358 (*p* < 0.01, *n* = 60) correlation between the infrared thermography imager and laser Doppler imager in 10 patients with scleroderma. At least one study (Lin and Saines, 2017) with eight patients with lower extremity arterial occlusive disease, the authors noted a difference in temperature distribution before and after undergoing endovascular intervention or a surgical bypass procedure. The improved flow was confirmed by arterial duplex ultrasound. Time series of thermographic images can be used for blood flow imaging and assessments. In such methods [see for example (Sagaidachnyi et al., 2017)], the temporal variations of the temperature can be used for the assessment of different physiological mechanisms, e.g., vasomotion. Signals in five frequency bands (0.005–0.02 Hz, 0.02–0.05 Hz, 0.05–0.15 Hz, 0.15–0.4 Hz, and 0.4–2.0 Hz) have endothelial (metabolic), neurogenic, myogenic, respiratory, and cardiac origins, respectively (Geyer et al., 2004). Taken together, the results demonstrate that thermography is a powerful tool to assess perfusion in the skin for diabetic subjects and people with other vascular conditions (Figure 4).

### Deep Tissue Injuries

When skin is subject to prolonged pressure or shearing forces, a pressure injury can occur. Although the terms decubitus ulcer, pressure sore, and pressure ulcer have often been used interchangeably, the National Pressure Injury Advisory Panel considers pressure injury (PI) the best term to use because open ulceration does not always occur (Table 1; Black et al., 2007). PIs are considered a preventable disease because the risk factors that lead to pressure injury can be identified in advance using tools like the Braden Score. Despite this, PI continues to occur in 6–18% of hospitalized patients (Li et al., 2020), with significantly higher prevalence in the chronically ill, patients in the intensive care unit, and those with spinal cord injury (Labeau et al., 2021). Given the considerable personal and financial costs the use of a practical method to detect early pressure injury is greatly needed.

**TABLE 1:** National Pressure Injury Advisory Panel (NPIAP) classification system.

Stage 1 pressure injury	Non-blanchable erythema of intact skin
Stage 2 pressure injury	Partial-thickness skin loss with exposed dermis
Stage 3 pressure injury	Full-thickness skin loss
Stage 4 pressure injury	Full-thickness skin and tissue loss
Unstageable pressure injury	Eschar obscured area which prevents depth determination
Deep tissue injury	Persistent non-blanchable deep red, maroon, or purple discoloration

The high incidence rate of PI calls for reliable and practical methods for early detection to guide therapeutic measures to mitigate progression. Inflammatory and apoptotic changes in the epidermal and dermal layers may precede surface changes by 3–10 days (Moore et al., 2017). In addition, clinical signs and symptoms which initially suggest a stage 1 PI may conceal a deep tissue injury. The tissue damage of deep tissue injury (DTI) in an area of intact skin evolves from the deep layers to the surface over 2–3 days and within 7–10 days further deterioration to a necrotic state occurs (Mayrovitz et al., 2018). The diagnosis of DTI is based on visual inspection alone, which is affected by subjective bias and user experience. In addition, the diagnosis can be exceedingly difficult in patients with dark skin (Fitzpatrick skin tone V–VI). More sensitive and accurate methods for detecting DTI are needed.

Risk assessment is the first step in the prevention of PIs. Unfortunately, the predictive value of current risk assessment scales is variable with modest performance (Adibelli and Korkmaz, 2019). A systematic review from Cochrane found no empirical evidence that using risk assessment scales can reduce the incidence rate of PI (Moore and Patton, 2019).

Infrared thermography offers the advantages of speed, simplicity, reproducibility and objective measurements. Numerous studies have reported thermal abnormalities occur with PI. Temperature measurements for skin assessments are recommended by international experts as part of the comprehensive skin and tissue assessment; the evidence-based statements published in the 2019 Clinical Practice Guideline include (Kottner et al., 2019; Internationalguideline, 2021):

(a) “Assess the temperature of the skin and soft tissue.”
(b) “When assessing darkly pigmented skin, consider the assessment of skin temperature and subepidermal moisture as important adjuncts assessment strategies.”

**FIGURE 5:**
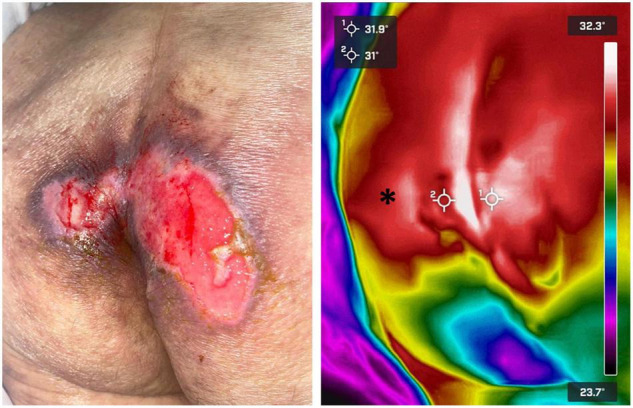
Pressure ulcer thermographic assessment. Infrared thermography was used to assess the severity of a sacral pressure ulcer in a bedridden patient. The wound’s temperature (crosshairs) was found to be very similar to the surrounding healthy skin (asterisk). Thermal gradients close to 0°C are highly suggestive of wounds that will re-epithelize by themselves in a relatively short term. Images, courtesy of Dr. Mario A. Martinez-Jimenez, were acquired using a FLIR One Pro camera.

Infrared thermography has been shown to be a useful physiological marker for assessment of PI development (Sae-Sia et al., 2005). Compared to normal skin, the site of injury may exhibit a rise or a fall in temperature (Figure 5).

**FIGURE 6:**
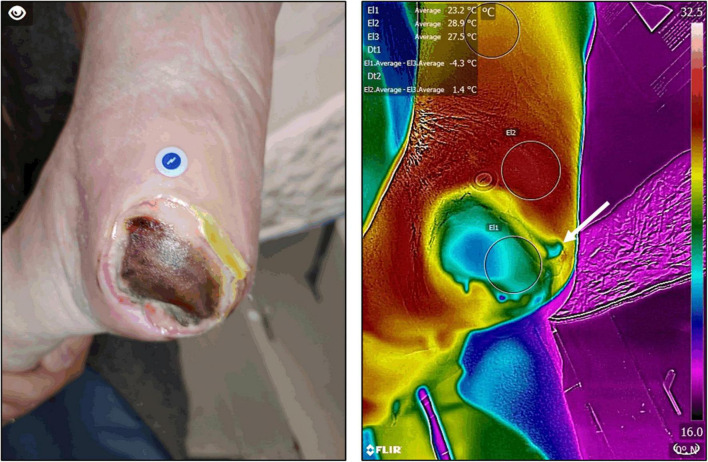
Deep tissue injury thermographic assessment. Infrared thermography was used to assess the severity of deep tissue injury in a patient with a healing pressure ulcer. Thermography confirmed an extensive area of devitalized tissue underneath the wound’s eschar. In addition, it also allowed the identification of significant peri-wound inflammation and more devitalized tissue (arrow) which was not apparent at clinical inspection alone. Images, courtesy of JR-G, were acquired using the Skin and Wound mobile app (Swift Medical Inc., Toronto, ON, Canada) paired to a FLIR One Pro mobile camera.

Two studies deserve special mention. The study by Simman and Angel (2021) was a blinded prospective cohort study of 70 ICU patients. Participants had their sacral area and bilateral heels scanned with the thermographic device. Follow-up imaging took place throughout the patient’s stay (upon admission and 3, 7, 14, and 25 days after). Clinicians were blinded from the thermal images and all participants received standard of care for the prevention and treatment of wounds. The objectives were an assessment of thermography for the detection of non-visual physiologic changes of DTI that can precede the visual changes, minimizing the subjectivity and difficulty of the visual assessment, and evaluating the financial impact of implementing thermographic technology. Of the 70 participants enrolled in this study, four patients with intact skin progressed to visually identifiable DTI’s. On all of these individuals, the thermographic device identified a pre-visual temperature anomaly before becoming a visually identifiable DTI. Three had an increased temperature (>1°C) above normal skin and one had a decreased temperature (−5.3°C) below average regional skin values. These temperature changes preceded the visual appearance of the DTI by 5–18 days (Figure 6).

The second notable study by Cai et al. (2021) evaluated 415 ICU patients enrolled by a convenience sampling method. The risk of PI was assessed via Braden Scale and thermal images of the sacral area were obtained by an infrared thermal imager once a day for 10 days. The predictive effects of infrared thermography and the Braden scale on PI were compared by the receiver operating characteristic curve from which the optimal cut-off value of skin temperature for predicting pressure injury was determined. The authors found that the relative temperature of the sacral area was negatively correlated with the risk of PI. The efficiency of infrared thermography for diagnosing PI was better than that of the Braden Scale. Based on the relative temperature optimal cut-off value (−0.1°C), Kaplan–Meier curve and Cox proportional hazard regression model analysis showed the incidence of pressure injury with relative temperature below −0.1°C was higher than the group with relative temperature above −0.1°C.

### Other Chronic Wound Infections and Inflammations

**FIGURE 7:**
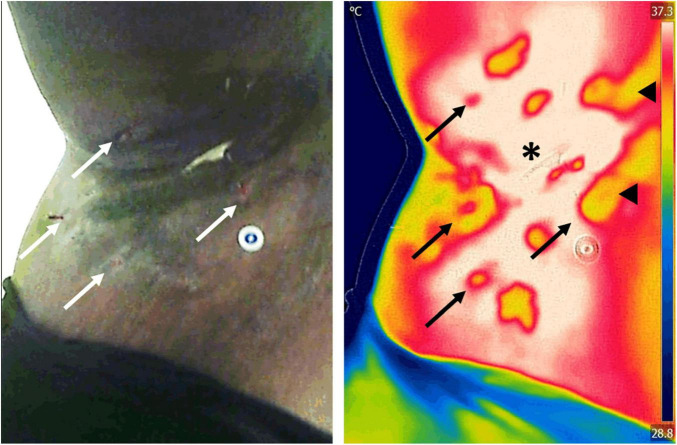
Assessment of inflammation in a patient with hidradenitis suppurativa. Infrared thermography was used to assess the severity and area of inflammation in a patient with hidradenitis suppurativa. Assessment of the area of inflammation on regular clinical photographs (left) is challenging in patients with darker skin tones, which leads to subjective patient severity scoring. In contrast, because infrared thermography images (right) are impervious to skin color, hotspots (asterisk) can easily be used to map the extent of inflammatory changes. Previous research has demonstrated that the area of inflammation and its temperature gradient highly correlate with current clinical scores, offering a powerful insight into the underlying condition. Furthermore, thermography is able to identify open wounds (arrows) and tunneling in the dermis (arrowheads) that may not be evident to clinical inspection alone. Images, courtesy of Dr. Sheila C. Wang and JR-G, were acquired using the Skin and Wound mobile app (Swift Medical Inc., Toronto, ON, Canada) paired to a FLIR One Pro mobile camera.

In the context of skin inflammatory disorders, infrared thermography has proven useful in detecting inflammation and staging the severity of the disease. For example, a positive temperature gradient of 2–4°C was found in the axillae and groins of people suffering from hidradenitis suppurativa, an inflammatory disease of the hair follicles (Zouboulis et al., 2019). Two recent studies demonstrated that in the same disease, when quantified, the positive gradient temperature has a high degree of correlation with several of its clinical staging systems (Ramirez-GarciaLuna J. et al., 2021; Ramirez-GarciaLuna J. L. et al., 2021). Due to the subjective nature of these scoring systems, the authors of these reports recommend using thermography as an objective tool to monitor disease severity and assess response to treatment during clinical trials (Figure 7).

Thermography can be used for treatment planning and monitoring. In particular, a case report suggested that thermography of patients with hidradenitis suppurativa has value in planning the surgical excision of the diseased area and that once the inflamed tissue is removed, the areas of positive temperature gradient normalize over time (Polidori et al., 2017), or that the lesions observed can be mapped to other medical imaging modalities, such as magnetic resonance (Derruau et al., 2018). Likewise, thermography has been used to monitor treatment response in patients with psoriasis as normalization of temperature gradients is correlated with disease improvement and affection to joints (Castillo-Martínez et al., 2013; Capo et al., 2015). Finally, thermography has been extensively used to map microvascular involvement in systemic inflammatory diseases such as lupus erythematosus and scleroderma (Saygin et al., 2019). For example, thermographic imaging of the hands of subjects with systemic sclerosis has an overall accuracy of 74% for correctly identifying people suffering from this disease (Murray et al., 2009) and is highly predictive of the development of digital ulcers (Hughes et al., 2016).

Beyond skin diseases, thermography has also been found to be effective for the identification of systemic conditions. Thermographic imaging of the abdomen of adults with acute abdominal pain was found to have a diagnostic accuracy comparable to that of ultrasound imaging to diagnose appendicitis (Ramirez-GarciaLuna et al., 2020). The authors of this study demonstrated that temperature gradients between the right and left lower abdominal quadrants as little as 0.35°C are highly predictive of appendicitis. Thus, if these temperatures are assessed with lower sensitivity tools, such as digital thermometers, diagnosis may be missed (Steele, 1986). The abdominal temperature difference may be related to pain, which is associated with blood flow redistribution due to autonomic nerve activation. Indeed, evidence of thermal changes in the lacrimal carunculae have been found to appear in response to noxious stimuli (Kolosovas-Machuca et al., 2016). This research opens the door for using thermography as a non-invasive imaging modality for measuring pain and its associated physiological responses; thereby, lowering the intrinsic subjectivity of these experiences (Erel and Özkan, 2017). Finally, in the context of the ongoing COVID-19 pandemic, infrared thermographic assessment has been proposed as an alternative to screen people to identify facial inflammatory changes suggestive of infection (Martinez-Jimenez et al., 2021). In contrast, regular fever screening with digital thermometers has been found to be poorly predictive of infection in minimally symptomatic adults (McConeghy et al., 2020). However, further research needs to be carried out to fully demonstrate the effectiveness of thermal infrared screening for this application.

### Surgical Site Infections

**FIGURE 8:**
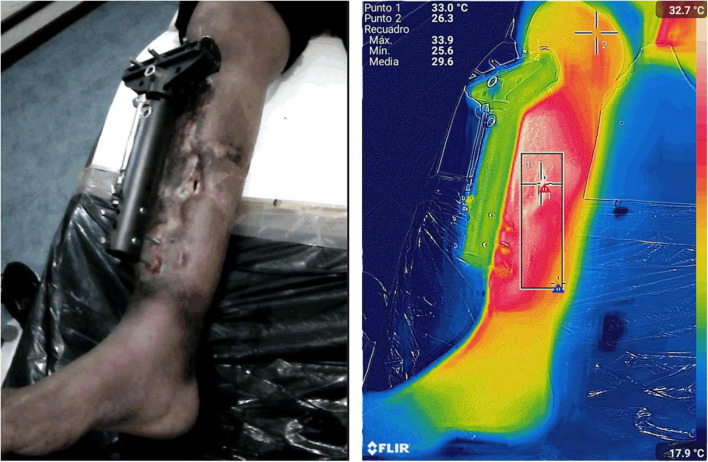
Assessment of a surgical site infection. Infrared thermography was used to monitor the extent of a surgical site infection following the external reduction of an open tibial fracture. The thermogram shows an area of extensive inflammation with the maximum thermal signal in the region of interest (ROI) bounded by the box. Images, courtesy of JR-G, were acquired using the Skin and Wound mobile app (Swift Medical Inc., Toronto, ON, Canada) paired to FLIR One Pro camera.

It is estimated that in North America alone, up to 30% of surgical patients develop a surgical site infection (SSI; SSI et al., 2021). SSIs are defined by the United States Centers for Disease Control (CDC) as the presence of purulent exudates, a systemic fever of more than 38°C, site pain, tenderness, localized swelling, redness or heat within 30 days after surgery (Horan et al., 2008). These definitions are very broad and non-specific, objective measurements to classify and diagnose patients with SSI are needed. Thermography has shown promise to be such an objective tool (Figure 8). For example, a study found differences in the thermal pattern in response to injury in the surgical incision in days 1–4 after surgery in patients who underwent enterostoma closures (Siah et al., 2019). The authors of this paper found that non-infected post-surgical wounds showed an increase of approximately 1.5°C in the periwound area within the first 48 h after surgery. In contrast, infected wounds did not show this pattern and took almost 4 days to reach similar temperatures. Furthermore, the abdomen of patients with post-surgical infections showed “coldspots” during the first 4 days after surgery, which were highly suggestive of SSIs. Another study where follow-up of obese women who underwent cesarean sections was done at 7, 15, and 30 days postoperative found a similar pattern (Childs et al., 2019). A temperature gradient of −1°C between the periwound area and the abdomen at day 7 after surgery was associated with a 3-fold increase in the likelihood of having a SSI. The authors also found that after this time point, temperatures between patients with or without SSI were very similar; thus, concluding that the value of thermal imaging for diagnosing SSIs was in the first days after surgery. In contrast, thermographic monitoring of patients following total knee arthroplasties showed that a 1.6°C increase between presurgical temperatures and the peak temperature in the first 3 days after surgery was highly suggestive of septic arthritis (Romanò et al., 2012). Likewise, persistent increases in the periwound temperature after procedures involving sternotomies were found to be suggestive of infection in two studies (Robicsek et al., 1984; Fujita et al., 2013). Finally, a recent report showed that temperatures above 34°C in the pin sites of patients who underwent external fracture reductions had a sensitivity of 73%; specificity, 67%; positive predictive value, 10%; and negative predictive value, 98% to detect infection. Additionally, the authors of this report demonstrated that the intra-rater agreement accuracy for thermography was excellent (Rahbek et al., 2021). Taken together, these findings strongly suggest that thermography may be a viable tool to objectively detect early SSIs; however, they also highlight the variability of measurements for different surgical procedures and anatomic locations and calls for more studies to be performed. Nonetheless, because infrared thermography can easily be paired with mobile devices (Wang et al., 2017) and smartphone apps have been demonstrated to be promising technologies for SSI monitoring (Wang et al., 2020), it can be expected that in the near future these combined technologies can be leveraged for the early identification of SSIs.

### Flap Viability

**FIGURE 9:**
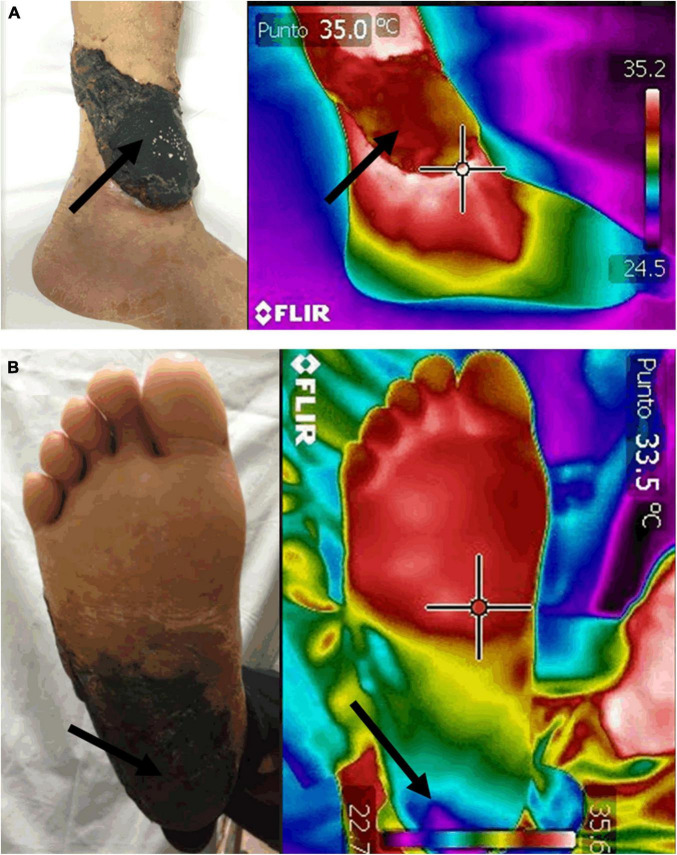
Assessment of free flap survival. Infrared thermography was used to assess survival of free tissue flaps (arrows) 2-weeks post-surgery. Restoration of the initial negative temperature gradient between uninjured tissue and the flap has been found to be highly predictive of flap perfusion and survival (A). In contrast, failing flaps show coldspots that represent areas of malperfusion (B). Interestingly, despite the clinical images of both patients showing dermal necrosis of the flaps, the thermal images show striking differences in tissue perfusion. Images, courtesy of Dr. Mario A. Martinez-Jimenez, were acquired using a FLIR One Pro mobile camera.

Flap loss is the most feared complication following flap surgery. Traditionally, flap monitoring is performed by clinical assessment, including the assessment of the tissue coloration, temperature, capillary refill and turgor (Shen et al., 2021). However, it is estimated that between 8 and 14% of cases of flap reconstructions present some degree of failure due to thrombosis of the tissue. If thrombosis is recognized promptly, the tissue may be salvaged; however, due to the subjective nature of clinical inspection alone, this does not always occur (Brands et al., 2010). Infrared thermography may be an attractive technology for measuring flap viability after surgical reconstruction because there is a direct relationship between tissue perfusion and its temperature, (de Weerd et al., 2011; Sagaidachnyi et al., 2017). One study found that hotspot images on infrared imaging after flap surgery were always associated with arterial Doppler signs over them (Sjøberg et al., 2020). Another study found that free flap failure was correlated with negative temperature gradients during surgery (−2.64°K), at 24 h post-operative (−1.22°K), and 10 days after surgery (−0.058°K) (Just et al., 2016). Similarly, Meyer et al. (2020) found that intraoperative non-rewarming of the tissue after surgical microvascular grafting was associated with flap loss. Before anastomosis, the mean temperature of flaps showed a negative temperature gradient of −11.47°F which was normalized to 0°F when the flow was adequately restored. Taken together, these findings strongly suggest that infrared technology can be used during flap surgery to predict patient outcomes (Figure 9). Indeed, infrared imaging has previously been used to predict the healing outcome, the need for surgical treatment, and the time needed to achieve wound healing in patients with burns (Martínez-Jiménez et al., 2018a; Carrière et al., 2020). However, due to the scarcity of scientific reports, caution for extending the conclusions on flap survival is warranted.

### Vascular Surgery

**FIGURE 10:**
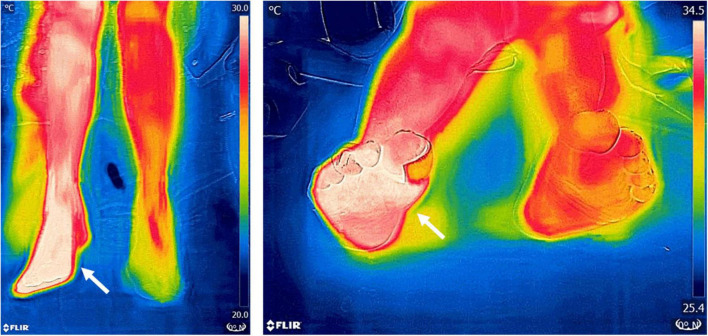
Thermographic imaging of a patient after endovascular revascularization. Thermograms 24 h post-surgery of a patient with severe peripheral artery disease (PAD) who underwent endovascular revascularization show a positive gradient of temperature of 4.2°C compared to the non-revascularized limb. The hotspots (arrows) are highly predictive of a successful treatment. In this patient, reperfusion was confirmed angiographically during surgery and by Doppler ultrasound at follow-up. Images, courtesy of JA-C, were acquired using a FLIR One Pro mobile camera.

Angiographic studies demonstrate that the skin’s temperature correlates directly with overall perfusion (Nagase et al., 2011). Thus, not surprisingly, patients with peripheral artery disease (PAD) exhibit disparities in their skin temperature distribution, particularly in the soles (Ilo et al., 2020). This finding was used to develop a prognostic measurement of vascular compromise and cardiovascular risk akin to the widely known ankle-brachial index (ABI). The thermographic ABI (tABI) consists of obtaining thermograms of the hands and feet of patients and dividing the peak lower extremity temperature by the upper extremity temperature (Wallace et al., 2018). The researchers who developed the method found a Pearson correlation of 0.83 (*p* < 0.001) between ABI and tABI measurements. Because this measurement is non-contact and non-invasive, it could be an attractive alternative to traditional ABI measurements if validated in larger patient cohorts. Likewise, the 6-min walk test (6MWT), another classic non-invasive study for the assessment of PAD, has been studied in a thermographic protocol with measurements before and after the patients got on the treadmill (Huang et al., 2011). The post-exercise temperature was found to drop in the lower extremities of patients with arterial stenosis, but they were maintained or elevated slightly in the extremities of people with patent arteries (temperature changes at sole in PAD vs. non-PAD patients: −1.25 vs. −0.15°C; *p* < 0.001). The exercise-induced temperature changes at the sole were also positively correlated with the 6MWT distance walked (Spearman correlation coefficient = 0.31, *p* = 0.03) and correlated with the ABI (Spearman correlation coefficient = 0.48, *p* < 0.001). Thus, the investigators of this study concluded that sole measurements after completing the walk had a sensitivity of 81.7% and specificity of 65% to diagnose PAD. Finally, successful revascularization of limbs with critical ischemia has demonstrated a classic thermographic pattern characterized by a significant increase in the temperature of the revascularized limb, compared to the contralateral side within the first 24 h after surgery (Staffa et al., 2017; Ilo et al., 2021; Figure 10). Thus, the plantar temperature has been used to predict amputations after endovascular therapy in this patient population with a sensitivity of 70% and specificity of 54% (Chang et al., 2020).

In the field of venous pathology, infrared thermography has been used to assess wound healing, response to treatment, and predict infection in patients with venous ulcers in a similar manner to patients with diabetic ulcers (Martínez-Jiménez et al., 2013, 2018b; Dini et al., 2015; Cwajda-Białasik et al., 2020). Finally, infrared thermography has also been used to predict fistula maturation in a cohort of 100 patients with end-stage renal disease who needed vascular access for hemodialysis (Al Shakarchi et al., 2017). Because the creation of an arterio-venous fistula (AVF) creates significant hemodynamic changes to blood flow in the arm, the authors of this report found that in turn, these changes lead to thermal changes distally. Negative temperature gradients between the patients’ hands were highly predictive of a successful AVF, while no changes or positive gradients almost always were associated with procedure failure. The authors of this report also found that for AVF clinical patency, infrared thermal imaging had a positive predictive value of 88% and a negative predictive value of 86% and that for functional maturation, it was 84 and 95%, respectively. Furthermore, they also concluded that thermography was highly superior to the commonly used intra-operative predictor of the presence of thrill or other independent pre-operative patient factors. Taken together, these reports strongly suggest that infrared thermography is an imaging modality with significant value in vascular surgery.

### Sports Medicine

Like all mammals, humans exhibit a wide degree of adaptive physiological changes to exercise, and because many of them entail thermoregulation, they can be readily assessed using thermal imaging (Mota-Rojas et al., 2021). As opposed to the previous use cases discussed in this review which have been done using static thermal imaging, the assessment of physiological responses to exercise requires dynamic imaging to capture the changes produced (Hillen et al., 2020). The studies that assess thermoregulation during exercise can be divided into three types: (a) constant long endurance exercise (more than 10 min with constant intensity), (b) resistance exercise (less than 10 min, typical resistance exercise with load), and (c) incremental exercise testing (increasing intensity protocol through exhaustion). In the first group, endurance exercise, common findings have been a blood flow restriction to the skin that produces a decrease in its temperature at the beginning of the exercise period followed by a rise of the skin temperature to basal or slightly above basal immediately after termination of the activity, with an in-between thermal plateau. This thermographic pattern reflects the redistribution of the blood flow to muscles during exercise and back to the skin after it to promote heat dissipation (Zaïdi et al., 2007; Balci et al., 2016; Drzazga et al., 2018; Hillen et al., 2020). In contrast, resistance exercise produces an increased skin temperature pattern related to the stressed muscles which is more pronounced in trained athletes, compared to untrained individuals, and in younger people compared to older individuals (Weigert et al., 2018; dos Santos Bunn et al., 2020). These findings can be linked to local and systemic adaptations of myocutaneous blood flow and reflect the muscles’ metabolism while under stress, as they have also been seen in individuals experiencing ergonomic injuries (Alexandre et al., 2017). Finally, for incremental exercise testing, a mixed pattern with no thermal changes at mild exercise levels and a similar pattern to endurance athletes of heat dissipation at the end of the workout session was observed at the highest intensity levels (Priego Quesada et al., 2017; Drzazga et al., 2018). These changes have been proposed as an adaptive response to heat dissipation of the core temperature, which is considered one of the most limiting factors for exercise performance (Lahiri et al., 2012; Hillen et al., 2020). Taken together, the findings strongly suggest that thermal imaging of athletes can be highly predictive of performance. Thus, it is not surprising that research using near-infrared imaging (NIR), an imaging modality closely related to thermal imaging but that can also measure oxygen content and that can be integrated into clothing materials, is a promising technology to assess athletes’ fitness, cardiovascular performance, ease of recovery, and predict injury risk (Bontemps et al., 2021).

## Discussion: The Future of Thermography

Here, we have presented an overview of the principles of infrared thermography, how this technology is sensed and detected by the current tools and highlighted some of its clinical applications in wound care, surgery, and sports medicine. Clinical thermography provides significant diagnostic value and prognostic insight to a wide range of clinical problems. This literature review has identified two primary use scenarios for thermographic imaging: inflammation-based and perfusion-based. These scenarios rely on local (topical) temperature measurements, which are different from systemic (core body temperature) measurements. Quantifying a disease requires thermographic imaging of an area in contrast to single-point measurements. For example, the severity of inflammatory disease is proportional to the total heat load which is a function of temperature and area.

The value of infrared thermography is a function of one’s perspective. In terms of efficiency, safety, and cost it is an attractive addition to the clinical toolbox. It is non-invasive, with fast acquisition times, and can provide simultaneous point measurements or average large area measurements. Maxim et al. (2014) observed that “As science increases its hold on the practice of medicine, we become more aware of the limitations of the clinical method.” A principal challenge with clinical methods is inter-observer variability and subjective bias. For many diseases, thermography serves as a screening tool that serves to clarify the differential diagnosis (clinical hypotheses) by confirming or disconfirming a disease process.

In its simplest form, the screening test has only two outcomes: positive (suggesting the disease is present) or negative (suggesting the disease is absent). An ideal screening test would have a positive result if and only if the patient has the disease (no false positive) and a negative result if and only they do not (no false negative). However, in clinical practice, screening tests understandably fall short of this clear dichotomy. This brings us to a current limitation of thermography for many disease processes which is Real World Data (RWD).

In clinical practice, it is important to consider efficacy versus effectiveness. Efficacy is “proof of principle” in a highly controlled environment with a uniform group of subjects and a single experienced interpreter of the thermography image. In contrast, effectiveness answers the question of test performance in everyday practice where the environment is uncontrolled, the patient population has numerous comorbidities, many different drug regimens, and multiple interpreters of the thermogram. In brief, efficacy is a controlled bench study with very little “noise.” Effectiveness is the uncontrolled clinical arena with substantially more “noise.”

Fortunately, the issue of test performance in clinical practice can be sorted out by using large digital data sets. The use of big data helps to provide clear confidence limits for test performance (prediction). Additionally, large data sets lend themselves to machine learning to identify new patterns and relationships within the data set. So far, thermography has demonstrated “proof of principle” but is lacking in large studies which provide the predictive confidence intervals needed to expand its adoption in practice. For machine learning to occur very large data sets are required. The size of the data set is partly dependent on the number of degrees of freedom in the proposed model. For healthcare one can reasonably assume data sets of 100,000 measurements or more from a diverse group of patients. In closing, the future of thermography is dependent on big data and refined confidence intervals for its predictive value in a variety of disease processes.

That being said, it is clear that clinical thermography is no longer an experimental clinical modality. There is growing confidence in the technology because there are a sufficient number of cross-validating studies with similar conclusions. As such, it is a technology that is becoming mature for a wider clinical application. The commercialization of thermography has made the technology more user-friendly which facilitates its migration from the bench to the bedside. However, the wide adoption of the technology would be accelerated by larger studies supporting the clinical utility of thermography. Availability of commercial modalities with cloud-based data will likely make it possible to generate RWD in the near future, which will then close the existing gaps in clinical data and open a wide array of new opportunities.

## Author Contributions

JR-G, RB, JA-C, RF, and GS contributed equally and involved in the draft preparation. All authors involved in the final revision.

## Conflict of Interest

JR-G is employed by Swift Medical Inc., as Director, Clinical Research and Validations. RB is employed by Swift Medical Inc., as Chief Medical Officer. RF is employed by Swift Medical Inc., as Director of Clinical Services – Canada. GS is employed by Swift Medical Inc., as VP Strategic Innovations. The remaining author declares that the research was conducted in the absence of any commercial or financial relationships that could be construed as a potential conflict of interest.

## Publisher’s Note

All claims expressed in this article are solely those of the authors and do not necessarily represent those of their affiliated organizations, or those of the publisher, the editors and the reviewers. Any product that may be evaluated in this article, or claim that may be made by its manufacturer, is not guaranteed or endorsed by the publisher.

## Abbreviations

6MWT: 6-minute walk test
ABI: Ankle-brachial index
AVF: Arterio-venous fistula
CDC: US Centers for Disease Control
DFI: Diabetic foot infection
DFU: Diabetic foot ulcer
DTI: Deep tissue injury
FPA: Focal plane array
ICU: Intensive care unit
IR: Infrared
MWIR: Middle wavelength infrared spectral range
LWIR: Long wavelength infrared spectral range
PA: Pulmonary artery
PAD: Peripheral artery disease
PI: Pressure injury
RWD: Real world data
SSI: Surgical site infection.
